# Secreted protein acidic and rich in cysteine (SPARC) induces apoptosis of human brain vascular smooth muscle cells through regulating HK2 in intracranial aneurysm

**DOI:** 10.3389/fnmol.2023.1290556

**Published:** 2023-11-23

**Authors:** Donglin Zhou, Tao Li, Xianjun Tan, Chenping Yun, Peng Jiang, Tongfu Zhang, Hong Kuang, Yunyan Wang

**Affiliations:** ^1^Department of Neurosurgery, Qilu Hospital of Shandong University, Cheeloo College of Medicine and Institute of Brain and Brain-Inspired Science, Shandong University, Jinan, Shandong, China; ^2^Department of Neurosurgery, The Third Affiliated Hospital of Shandong First Medical University, Shandong First Medical University, Jinan, China; ^3^Department of Neurosurgery, Chiping District Traditional Chinese Medicine Hospital, Liaocheng, China; ^4^Shandong First Medical University and Shandong Academy of Medical Sciences, Jinan, China; ^5^Jiangsu Center for the Collaboration and Innovation of Cancer Biotherapy, Cancer Institute, Xuzhou Medical University, Xuzhou, China; ^6^Department of Neurosurgery, Yangxin County People’s Hospital, Binzhou, China; ^7^Department of Neurosurgery, The Second Affiliated Hospital of Guangxi Medical University, Nanning, Guangxi, China

**Keywords:** SPARC, apoptosis, HK2, intracranial aneurysm, human brain vascular smooth muscle cell

## Abstract

**Background:**

Vascular smooth muscle cell (VSMC) dysfunction is one of the crucial pathologic processes in the development of intracranial aneurysm (IA). Secreted protein acidic and rich in cysteine (SPARC), a multifunctional glycoprotein, is overexpressed in many tumor, but its underlying mechanism in vascular disease has not been elucidated. The aim of this study is to evaluate the potential function of SPARC in IA generation and regulation of mitochondrial function in VSMC.

**Methods:**

Human brain vascular smooth muscle cells were treated with recombinant SPARC to detect apoptosis-related markers. The downstream targets affecting mitochondrial dysfunction after SPARC treatment were explored by transcriptome sequencing and bioinformatics analysis, and verified using by immunohistochemistry and western blot. Further *in vitro* experiments verified the role of downstream targets in regulating VSMC mitochondrial function.

**Results:**

Secreted protein acidic and rich in cysteine (SPARC) expression was associated with the risk of IA rupture. SPARC induces mitochondrial pathway apoptosis in human brain VSMC. We screened 40 differentially expressed genes related to mitochondrial function after SPARC treatment. Hexokinase 2 (HK2) was identified as a downstream target of mitochondrial pathway apoptosis in VSMC induced by SPARC. In addition, immunohistochemical results confirmed that the difference between SPARC and HK2 expression is located mainly in the smooth muscle layer of IA. Overexpression of HK2 reversed the SPARC-induced increase in apoptosis and mitochondrial damage in VSMC.

**Conclusion:**

Secreted protein acidic and rich in cysteine (SPARC) regulated mitochondrial function in VSMC and induced apoptosis through HK2, which plays an important role in the formation and rupture of IA. Targeting SPARC may be a novel strategy to delay the development of intracranial aneurysms.

## Introduction

Intracranial aneurysm (IA) is considered as a form of cerebrovascular degeneration that is characterized by tumor-like protrusion formed by local abnormal dilation of intracranial artery vessels and thinning of the tube wall. The pathophysiological mechanisms of IA is highly complicated, but it mainly includes the breakdown of arterial homeostasis, degradation of extracellular matrix (ECM) and apoptosis of vascular smooth muscle cells (VSMCs) (Kamio et al., 2018; Liu et al., 2018). The ECM and smooth muscle layer are important building blocks for maintaining the strength of the vessel walls, and dysfunction of VSMCs has been widely recognized as a key link in the occurrence and rupture of IA. Hara et al. (1998) observed a large number of apoptotic cells in ruptured IA tissue, with a higher density of apoptotic cells near the rupture site. The disordered arrangement and decrease of VSMCs in IA wall have also been confirmed in animal experimental models, which is partly resulted from VSMCs apoptosis (Frosen, 2014). Furthermore, inflammation, oxidative stress, mechanical stress of abnormal blood flow dynamics and other factors can induce apoptosis of VSMCs, thereby reducing the strength of blood vessel wall (Frosen et al., 2012).

Secreted protein acidic and rich in cysteine (SPARC) is first identified in renal cysts and tumors. This protein has a wide range of biological functions involved in the occurrence of tumors, vascular diseases and metabolic diseases, for instance, regulating ECM remodeling, cell adhesion, and angiogenesis (Cheetham et al., 2008; Rosset and Bradshaw, 2016). The role of SPARC in vascular endothelial cells and VSMCs has received increasing attention. Study found that the expression level of SPARC in IA is significantly increased, which is correlated with the production of matrix metalloproteinase 2 and 9 (MMP-2/9) (Li et al., 2013). This indicates that SPARC is closely related to the occurrence of IA, but the specific molecular mechanisms involved in its pathogenesis are unclear. Previous studies confirmed that exogenous SPARC stimulation can induce autophagy of human brain vascular smooth muscles (HBVSMCs) and promote the phenotypic transformation from systolic to synthetic. Moreover, increased expression of MMP-9, MCP-1 and other pro-inflammatory factors aggravates the progression of apoptosis (Ye et al., 2016; Li et al., 2019; Tan et al., 2020). SPARC also up-regulates the expression level of cleaved caspase 3 and cleaved poly ADP-ribose polymerase, suggesting the initiation of mitochondrial pathway apoptosis (Sailaja et al., 2013). The present findings indicated that SPARC may induce apoptosis in HBVSMCs via the mitochondrial pathway but this process remains to be fully explored.

This research analyzed the whole genome expression profiles of HBVSMCs treated with exogenous SPARC, and performed bioinformatics to analyze the assay results. We intersected the identified differentially expressed genes (DEGs) with mitochondria-associated genes to determine mitochondria-associated differentially expressed genes (DEMRGs). Protein-protein interaction (PPI) and gene ontology (GO) enrichment analyses revealed the functions and interactions of these genes. Specifically, HK2 was a significantly down-regulated gene associated with the regulation of mitochondrial function. In this study, we demonstrated that SPARC significantly down-regulated HK2 expression, and its interference with HK2 expression resulted in apoptosis and declined mitochondrial membrane sites. HK2 was considered as a downstream target of SPARC-induced apoptosis in the mitochondrial pathway of HBVSMCs.

## Materials and methods

### Cell culture and transfection

Primary HBVSMCs were purchased from ScienCell Research Laboratories (No. 1100, Carlsbad, CA), the 5th passage of the cells was used in this study. HBVSMCs were cultured in smooth muscle cell medium (SMCM, Cat.No. 1101, ScienCell Research Laboratories, Carlsbad, CA) with 5% fetal bovine serum (FBS, Cat.No. 0010), 1% smooth muscle cell growth supplement (SMCGS, Cat.No. 1152), and 1% penicillin/streptomycin (P/S, Cat. No. 0503) in a humidified incubator with 5% CO2 at 37°C. Complementary DNA (cDNA) targeting human HK2 (NM_00189) and small interfering RNA (siRNA) were synthesized (GenePharma, Shanghai, China). Lipofectamine™ 2000 mix (Invitrogen, Carlsbad, United States) and cDNA or siRNA transfection mix were prepared according to the instructions and let stand for 15 min. The above prepared transfection mix was added to each well, gently shaken, and placed in cell culture incubator for another 6 h. The complete SMCM medium containing fetal bovine serum was changed to continue incubation for 24 h before subsequent experiments. Sequences of siRNAs were listed in Supplementary Table 1.

According to the manufacturer’s protocol, SPARC (Cat.#Z02804, Genscript, Jiangsu, China) powder was dissolved in the matching dissolution and dilution buffer to obtain 100 ug/mL stock solution. The stock solutions were kept in aliquot at −20°C and immediately thawed before each experiment.

### Western blot

The total proteins of cells were extracted using Total Protein Extraction Kit (BestBio, Shanghai, China), according to the manufacturer’s instructions. The samples were separated by electrophoresis using 10–12% SDS-PAGE and then transferred to PVDF membranes and blocked with 5% skimmed milk for 1 h. Primary antibodies were added to the membranes at 4°C overnight. After washing three times with washing buffer, the membranes were incubated with a secondary antibody at 37 °C for 1 h. Enhanced chemiluminescence (ECL) was utilized for protein detection. The images were analyzed using a Molecular Imager Chemidoc XRS System (Bio-rad Laboratories, Hercules, CA, USA). The band abundance was calculated using Imange J software. All the primary antibodies used in this study were listed in the Supplementary Table 2.

### Cell apoptosis assay

Apoptotic cells were determined using Annexin V-AbFluor™ 488/PI apoptosis Detection Kit (Cat. #KTA0002, Abbkine, Wuhan, China), according to the manufacturer’s protocol. Briefly, single-cell suspension was incubated with 5 μl annexin V and 2 μl PI for 15 min at room temperature. The cells were analyzed by flow cytometry (NovoCyte, Agilent, USA). A total of 10,000 events were recorded for each analysis using FlowJo software.

### Hoechst 33342/PI double staining

Apoptosis and Necrosis Detection Kit was purchased from Beyotime Biotechnology. 0.5 mL of cell staining buffer per well was added to the well, followed by adding 5 μL of Hoechst 33342 and 5 μL of PI staining solution, respectively. The well was gently shaken and incubated for 30 min at 4°C in the dark. After staining, HBVSMCs were placed under an inverted fluorescence microscope for observation and taking pictures.

### Transmission electron microscopy (TEM)

The cells were collected by centrifugation, added with 2.5% glutaraldehyde was added, and fixed at 4°C for 2 h. Phosphoric acid buffer (0.1 M) was added to the cells and centrifuged. The precipitate was fixed with 2.5% glutaraldehyde at 4°C for 2 h. After ethanol gradient dehydration at 4°C and acetone gradient dehydration at room temperature, anhydrous acetone was soaked for 4 h and then embedded. Finally, the sections were stained with 4% uranium acetate for 20 min and lead citrate for 5 min and then placed under a JEM-100cxII electron microscope for observation.

### MMP Detection Kit (JC-1)

JC-1 was purchased from Beyotime (Cat. #C2006, Shanghai, China). Cell climbing tablets were prepared, washed with PBS and then 1 ml of medium and JC-1 working solution were added to each well and incubated in an incubator for 20 min. The supernatant was removed, after washing the cells twice using JC-1 staining buffer, 2 ml of medium was added. HBVSMCs were observed under a laser confocal microscope (Carl Zeiss, Germany, Zeiss LSM780, Oberkochen, Germany). The cells loaded with JC-1 were excited at 488 nm, and emission was detected at 590 nm (red, JC-1 aggregates) and 525 nm (green, JC-1 monomers). The levels of MMP in different groups were reflected by the ratio of green/red fluorescence.

### RNA extraction and RNA-Seq

Extraction of total RNA was performed using TRIzol Reagent (Invitrogen, Carlsbad, CA, USA). The appropriate concentration was determined by measuring absorbance at 260 nm using a Nanodrop spectrophotometer (Thermo Fisher Scientific, Waltham, MA, USA). RNA samples were prepared as described above. We entrusted Shanghai Genechem Company for chip detection on total RNA sample, and the Human Clariom™ S Assay expression microarray gene chip was used to detect the whole gene expression profile. The whole transcription chip information was analyzed and processed.

### Identification of differentially expressed mitochondrial-related genes (DEMRGs)

A total of 1,154 mitochondrial-related genes were obtain in a total of 87 gene sets selected from the GSEA website.^1^ The “limma” package of R software was used to identify the DEMRGs. Genes with *P*-value < 0.05 and | log_2_ (Fold Change)| > 0.585 were considered as DEGs. The heatmap and volcano plot were generated using “pheatmap” (v1.0.12) and “ggplot2” (v3.4.2) packages of R software.

### PPI construction and functional enrichment of DEMRGs

Protein-protein interaction (PPI) analysis of DEMRGs was conducted using the Search Tool for the Retrieval of Interacting Genes (STRING) database v11.5.^2^ Interactions with a combined score ≥ 0.4 were considered as statistically significant. Subsequently, the PPI network was visualized using Cytoscape v3.8 software and the hub nodes were identified by a high score based on a scale-free network. The maximal clique centrality (MCC) algorithm in CytoHubba plug-in was performed to further explore hub genes. Then, GO pathway enrichment analysis consisting of biological process (BP), molecular function (MF) and cellular component (CC) was performed on the DEMRGs on the Database for Annotation Visualization and Integrated Discovery (DAVID) website. The results was visualized using SangerBox.^3^

### Tissue specimens and immunohistochemical (IHC) staining

The tissues of IA and superficial temporal arteries (STAs) from patients who underwent aneurysm clipping and STA-middle cerebral artery bypass at Qilu Hospital of Shandong University were collected. The study was strictly conducted according to the principles of the Declaration of Helsinki. The acquisition of tissue samples was approved by the Ethics Committee on Scientific Research of Shandong University Qilu Hospital. Informed consent was obtained from all the patients.

Paraffin sections were incubated with SPARC and HK2 antibodies overnight at 4°C after dewaxing and antigen repair. Then, the sections were incubated with rabbit anti-goat secondary antibodies labeled with corresponding horse radish peroxidase (Cell Signaling Technology, MA, USA) at 25°C for 30 min. Next, the sections were stained with 3,3’-diaminobenzidine, counterstained with hematoxylin (GeneTech, Shanghai, China) and then visualized. The staining distribution and intensity of positive cells were observed.

### Immunofluorescent (IF) staining

The cells were washed with PBS, treated with pre-cooled 4% paraformaldehyde, and fixed for 20 min. After removing the fixative solution removed, the cells were rinsed and added with 0.2% TritonX-100 to break the membrane at room temperature for 10 min. After blocked with 5% BSA blocking solution, the diluted primary antibody was added and shaken gently at 4°C overnight. Removing the primary antibody, the anti-rabbit Alexa Fluor555 secondary antibody (Cat. #A0453, Beyotime, Shanghai, China) was added to the cells and incubated together at room temperature for 1 h away from light. The nuclei were stained with 4,6-diamino-2-phenyl indole (DAPI) staining solution and placed under a confocal microscope for observation and photographing.

### Statistical analysis

The experimental data were expressed as mean ± standard deviation, and all the experiments were repeated more than three times. Statistical analysis was performed using SPSS 21.0 software. Student’s *t*-test was used for the two-group comparison, one-way ANOVA was used for the multi-group comparison. *P* < 0.05 indicated a statistically significant difference. Charting was performed in GraphPad Prism 6.0 software. Western blot bands for gray values and immunofluorescence staining results for fluorescence intensity were analyzed using Image J software.

## Results

### High expression of SPARC was associated with the risk of IA rupture

**FIGURE 1:**
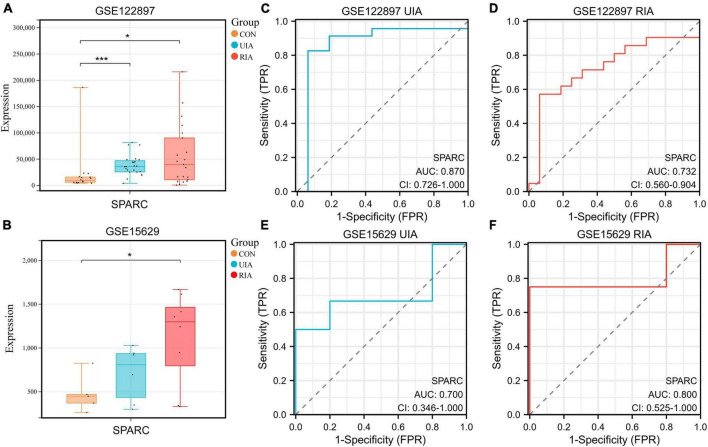
Expressions of SPARC in the database. **(A,B)** The boxplots showed the expression of SPARC in the control group, the unruptured group and the ruptured group of GSE15629 and GSE122897 databases. **(C–F)** ROC curves of SPARC in GSE15629 and GSE122897 databases. *P < 0.05, ***P < 0.001.

The IA datasets GSE15629 (Pera et al., 2010) and GSE122897 (Kleinloog et al., 2016) were successfully downloaded from the Gene Expression Omnibus (GEO) database using the “GEOquery” R package (Vargas et al., 2018) (v2.62.2). These two datasets contain control, unruptured and ruptured IA samples. The expression of SPARC was observed by plotting a box plot (Figures 1A, B). It was found that SPARC was remarkably increased in IA samples from both datasets, and its expression was higher in ruptured aneurysms than in unruptured aneurysms, suggesting that the high expression of SPARC may increase the risk of ruptured IA. We then constructed receiver operating characteristic (ROC) curves, indicating that SPARC has a better predictive ability for both the occurrence and rupture of IA (Figures 1C–F).

### SPARC induced apoptotic cell death in HBVSMCs

**FIGURE 2:**
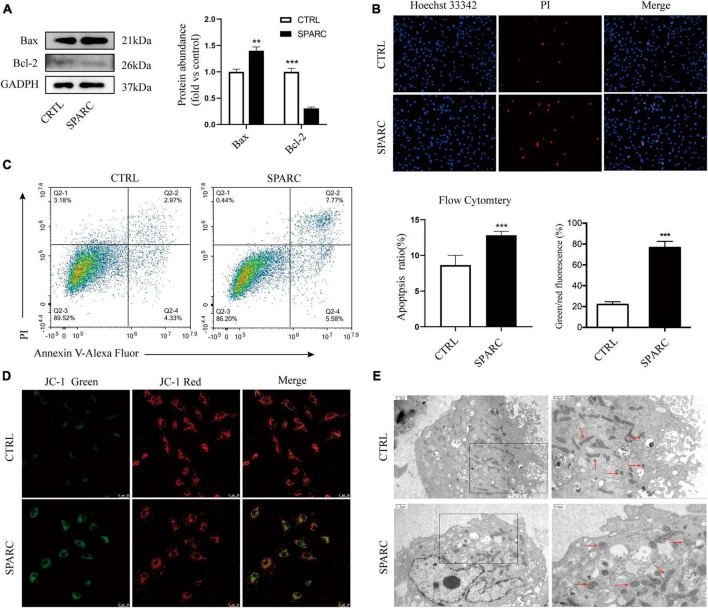
SPARC induced apoptosis of HBVSMC. **(A)** The protein levels of mitochondrial death pathway were determined by western blot assay. **(B)** Hoechst 33342/PI staining showed that the proportion of red and blue double staining cells increased, indicating cell apoptosis. **(C)** The proportion of apoptotic cells after SPARC (2 ug/mL) treatment for 24 h was measured by flow cytometry. **(D)** The changes of mitochondrial membrane potential of HBVSMCs without or with SPARC (2 ug/mL) for 24 h were visualized by JC-1 staining. **(E)** Transmission electron microscopy was used to observe the mitochondrial morphology of HBVSMCs treated with SPARC (2 ug/mL) for 24 h. The red arrow points to the mitochondria. **P < 0.01, ***P < 0.001.

Due to the exocrine properties of SPARC, our previous study treated smooth muscle cells with 0–4 ug/mL SPARC for 24 h to assess the effect of SPARC on cell viability. CCK-8 assay showed that SPARC inhibited the viability of smooth muscle cells in a concentration-dependent manner, and that the change of cell viability was statistically significant when SPARC concentration was 2 ug/mL (Ye et al., 2016). Hence, SPARC at 2 ug/mL was selected for subsequent experiments. Western blot results showed that SPARC could up-regulate the expression of pro-apoptotic proteins Bax but down-regulate the expression of anti-apoptotic protein Bcl-2 (Figure 2A). These proteins are commonly used to assess apoptosis in the mitochondrial pathway. After that, Hoechst 33342/PI staining primarily showed that the number of blue and red double-stained cells increased slightly in SPARC-treated HBVSMCs, suggesting the possible presence of apoptosis (Figure 2B). Annexin V/PI flow cytometry was performed to further determine the rate of apoptotic cells, and the results confirmed that SPARC treatment induced apoptosis process of HBVSMC, mainly early apoptotic cells (Figure 2C). Moreover, the detection of JC-1 mitochondrial membrane potential (MMP) also indicated that HBVSMCs changed from red light to green light after SPARC treatment, indicating the decrease of MMP and damage of mitochondrial function (Figure 2D). These results provided a new direction to explore apoptosis of HBVSMCs induced by SPARC through mitochondrial function damage pathway.

### SPARC treatment resulted in mitochondrial ultrastructural alterations in HBVSMCs.

We applied TEM to further observe the changes in mitochondrial ultrastructure of HBVSMCs after SPARC treatment for 24 h. The results showed that the mitochondrial volume of HBVSMCs in the control group was normal, round, or oval, with clear mitochondrial crest and mitochondrial membrane space and a homogeneous matrix. In the SPARC group, we observed that the mitochondria of HBVSMCs were significantly swollen, the mitochondrial crest was broken or even disappeared, the mitochondrial membrane space was not clear and the matrix became lighter (Figure 2E). These evidences fully confirmed that SPARC treatment may lead to serious damage to the mitochondria of HBVSMCs.

### Identification of DEMRGs

**FIGURE 3:**
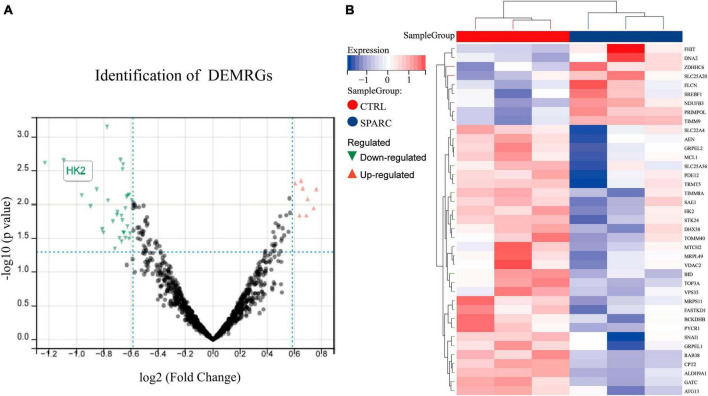
Identification of DEMRGs. **(A)** The volcano plot of the 40 DEMRGs. The *x*-axis was log_2_ (Fold Change), while the *y*-axis was -log (*P*-value). The red dots represent up-regulated genes and the green dots represented down-regulated genes, whereas gray dots represent non-significant expressed genes. *P*-value < 0.05 and | log_2_ (Fold Change)| > 0.585. **(B)** The heat map of 40 DEMRGs. Each row represents a gene and each column represents a sample.

To explore possible downstream mitochondrial mechanisms of SPARC-induced apoptosis in smooth muscle cells, we performed RNA-seq to investigate SPARC-induced transcriptomic alterations in HBVSMCs. The experiment consisted of SPARC groups (2 ug/mL SPARC treatment) and control groups, with 3 cases in each group. Mitochondria-related genes were selected from transcriptome data and analyzed. In total, 9 genes were consistently up-regulated in comparison to 31 consistently down-regulated genes by SPARC in HBVSMCs (Table 1). The expression of DEMRGs was visualized in volcano plot and heat map (Figures 3A, B).

**TABLE 1:** The 40 differential genes associated with mitochondrial dysfunction between SPARC treatment and control.

Gene symbol	Changes	Log(FC)	*P*-value
TIMM9	Up	0.60628267	0.00481668
SLC25A20	Up	0.63780167	0.01440887
PRIMPOL	Up	0.649291	0.00440286
NDUFB3	Up	0.66182533	0.00573378
FLCN	Up	0.688553	0.01440335
FHIT	Up	0.69850933	0.00822185
DNA2	Up	0.742154	0.01127191
ZDHHC6	Up	0.76191067	0.00590035
SREBF1	Up	0.82830833	0.02648283
STK24	Down	−1.2298443	0.00243084
HK2	Down	−1.0961537	0.00223194
BCKDHB	Down	−0.963033	0.00737366
MRPL49	Down	−0.898048	0.01068014
TIMM8A	Down	−0.8530303	0.0059992
PDE12	Down	−0.8117313	0.02345162
TRMT5	Down	−0.8029643	0.02632432
CPT2	Down	−0.7759887	0.00070895
FASTKD1	Down	−0.769064	0.00872967
SNAI1	Down	−0.7328543	0.01791122
HSP90AA1	Down	−0.7184743	0.04549663
AEN	Down	−0.7036207	0.0142414
VPS35	Down	−0.6998853	0.01301001
SLC22A4	Down	−0.6840633	0.03133267
RAB38	Down	−0.676543	0.00219024
GRPEL2	Down	−0.669861	0.01465037
VDAC2	Down	−0.668901	0.03553649
MCL1	Down	−0.663369	0.01717545
GATC	Down	−0.661805	0.00300658
ALDH9A1	Down	−0.657618	0.00255569
SAE1	Down	−0.6562447	0.02595473
SLC25A36	Down	−0.651404	0.02645639
TOMM40	Down	−0.641175	0.03102781
PYCR1	Down	−0.6408457	0.01137603
MRPS11	Down	−0.6323807	0.00771626
GRPEL1	Down	−0.6257603	0.02126532
BID	Down	−0.6237167	0.00740124
MTCH2	Down	−0.6143433	0.03202153
TOP3A	Down	−0.6133117	0.00719041
DHX30	Down	−0.606716	0.02691063
ATG13	Down	−0.5940107	0.00863048

### GO enrichment and PPI network of DEMRGs

**FIGURE 4:**
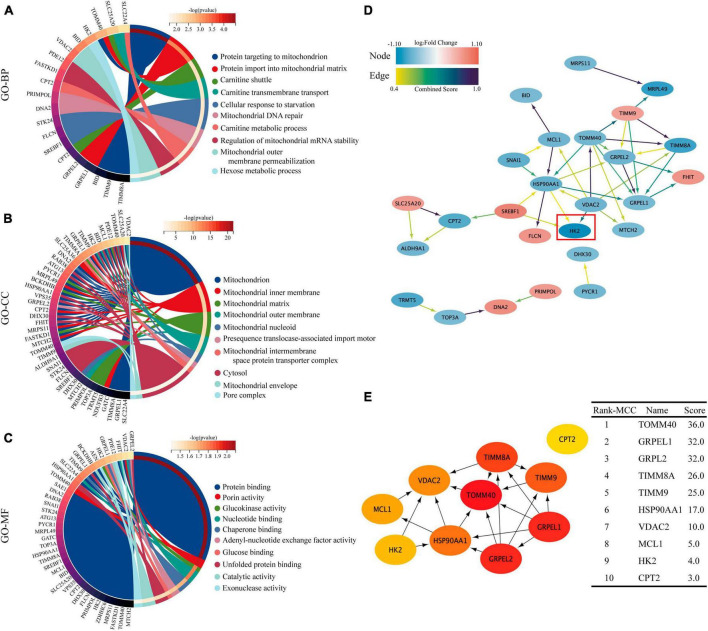
GO enrichment analysis and PPI network of 40 DEMRGs. **(A–C)** The chord plot showing the top 10 information of DEMRGs involved in each GO term. **(A)** Biological process (BP), **(B)** cellular components (CC), **(C)** molecular function (MF). **(D)** Using the STRING online database, the PPI network of 40 DEMRGs was constructed, including 26 nodes and 40 edges. The color of node represents log_2_ (Fold Change) value of gene encoding the protein and the color of edges represents Combined Score between two proteins. **(E)** The first 10 hub genes obtained using the MCC algorithm in the CytoHubba plug-in. The darker the color, the higher the score.

Gene ontology (GO) functional enrichment analysis revealed that these DEMRGs were mainly in mitochondrial membrane and matrix, and were mainly related to mitochondrial protein binding and energy metabolism, including carnitine shuttle and glucokinase activity pathway. We found that HK2 localized in the mitochondrial outer membrane and cytoplasm may be involved in protein binding, glucokinase activity, and glucose binding processes (Figures 4A–C). The PPI network developed using the STRING database revealed the interactions between the proteins encoded by the DEMRGs. This network was visualized by Cytoscape software and contained 26 nodes and 40 edges, with nodes representing genes and edges representing interactions between genes (Figure 4D). The top 10 genes were identified as hub genes according to the MCC algorithm using the CytoHubba plugin and ordered as follows: TOMM40, GRPEL1, GRPEL2, TIMM8A, TIMM9, HSP90AA1, VDAC2, MCL1, HK2, and CPT2 (Figure 4E). In this network, we observed that HK2 was the most obviously down-regulated mitochondria-related gene in these hub genes, and it was closely related to the interaction of VDAC2, HSP90AA1 and SREBF1. Follow-up experiments were then conducted to verify the association between SPARC and HK2 and the regulation of mitochondrial function.

### SPARC treatment inhibited HK2 expression in HBVSMCs.

**FIGURE 5:**
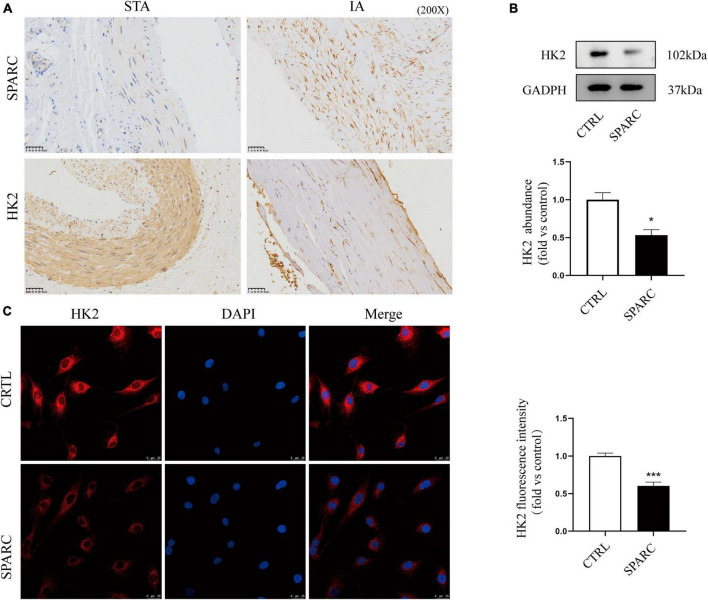
Validation of HK2 expression a IHC staining of STAs and IA. **(A)** The results indicated that the expression of HK2 in intracranial aneurysm wall was significantly reduced. Both HK2 and SPARC were localized in vascular smooth muscle layer. **(B,C)** The expression pattern of HK2 after SPARC (2 ug/mL) treatment for 24 h by western blot and immunofluorescence. These results suggested that the inhibition of HK2 expression may be related to SPARC. *P < 0.05, ***P < 0.001.

The chip results and the above analysis results showed that SPARC could induce significant down-regulation of HK2 expression in HBVSMCs, and that HK2 participated in multiple GO enrichment analysis pathways. We further explore whether HK2 played an important role in SPARC-induced mitochondrial damage. The results of IHC demonstrated that the distributions of SPARC and HK2 appeared to be mainly concentrated in vascular smooth muscle layer. Although the number of VSMCs in the aneurysm wall was reduced and the alignment was disordered, the expression of SPARC was increased and the expression of HK2 was significantly reduced compared to normal vascular tissue (Figure 5A). This suggested a potential correlation between SPARC and HK2 in smooth muscle cells, which was consistent with the results of chip analysis. We also verified that SPARC induced HK2 expression in HBVSMCs at the protein level by applying immunofluorescence staining and western blot. HK2 in the control group showed red color, strong fluorescence intensity, and staining near the nucleus was the most obvious but HK2 in SPARC group was obviously lightened, indicating that the expression of HK2 decreased (Figure 5B). We further analyzed HK2 protein expression of HBVSMCs after SPARC treatment. The results showed that the expression of HK2 in HBVSMCs decreased signally after SPARC treatment (Figure 5C). These evidences indicated that HK2 was the downstream target of SPARC on HBVSMCs and may participate in SPARC-induced mitochondrial pathway apoptosis.

### Silencing HK2 suppressed HK2 expression and mitochondrial function in HBVSMCs

**FIGURE 6:**
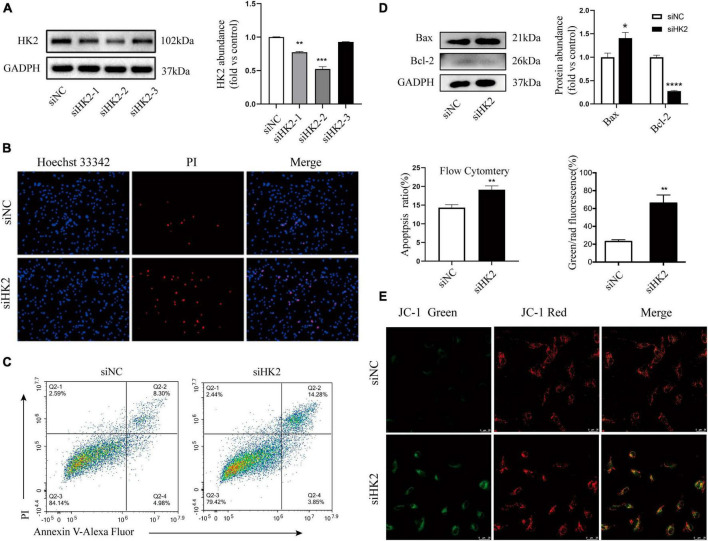
Silencing HK2 expression promotes apoptosis. **(A)** The protein levels of HK2 in siRNA transfected cells were detected by western blot. Since siHK2-2 has the highest knock-down efficiency, siHK2-2 is selected for subsequent experiments. **(B)** The cell status was observed by Hoechst 33342/PI staining. **(C)** Apoptotic cell frequencies treated without or with transfection were determined by Annexin V/PI assays. **(D)** The protein levels of Bax and Bcl-2 in siRNAs transfected cells were determined by western blot. **(E)** JC-1 staining suggested that HK2 silencing led to a decrease in mitochondrial membrane potential. *P < 0.05, **P < 0.01, ***P < 0.001, ****P < 0.0001.

Mitochondria-associated HK2 is not only associated with cell metabolism, but also regulates cell death (Kim et al., 2007; Chen et al., 2009). Three small interfering RNAs targeting HK2 were transfected into HBVSMC. Western blot assays showed that three small interfering RNAs targeting HK2 successfully reduced protein levels of HK2 (Figure 6A). Hoechst 33342/PI double staining showed that after transfection of siHK2, HBVSMCs had different nuclei sizes, divided nuclei, and the number of apoptotic cells stained with bright blue and red were significantly increased (Figure 6B). Flow cytometry apoptosis assay was performed to more accurately evaluate the increased apoptosis rate after HK2 silencing (Figure 6C). We also noted that silencing HK2 resulted in a consistent expression trend in Bax, and Bcl-2 after SPARC treatment (Figure 6D). Furthermore, based on these results, JC-1 staining was performed and confirmed the changes in MMP. The green/red fluorescence ratio increased in the siHK2 group compared with the control group (Figure 6E), indicating a decrease in MMP of HBVSMCs. Hence, our data suggested that silencing HK2 induced mitochondrial pathway apoptosis.

### Overexpression of HK2 reversed the apoptosis induced by SPARC

**FIGURE 7:**
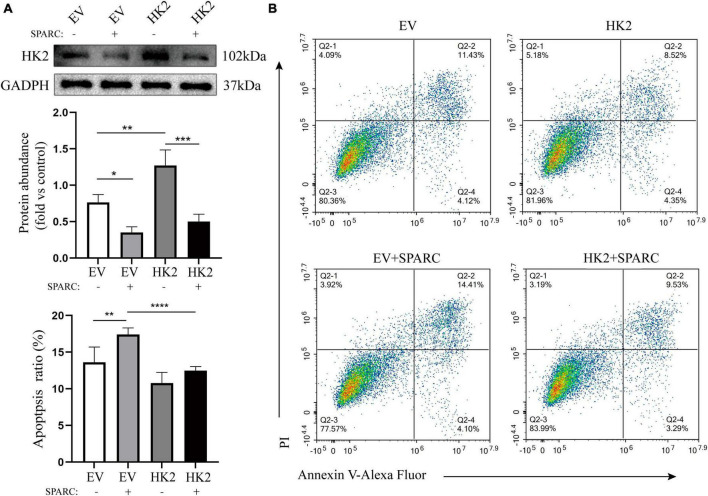
HK2 prevents SPARC-induced apoptosis. **(A)** Western blot was used to detect the expression level of HK2 in cells transfected with cDNA and overexpression of HK2 improved SPARC-induced down-regulation of HK2. **(B)** Overexpression of HK2 decreased the proportion of apoptotic cells induced by SPARC. *P < 0.05, **P < 0.01, ***P < 0.001, ****P < 0.0001.

We transferred HK2-targeting cDNA into HBVSMCs. Western blot assay showed that successful transfer of overexpressed plasmid increased the expression of HK2 and reversed down-regulated HK2 induced by SPARC (Figure 7A). Flow cytometry also demonstrated that HK2 overexpression could improve the apoptosis of SPARC-induced HBVSMC. This further indicated that SPARC targeting HK2 caused apoptosis (Figure 7B).

## Discussion

This study developed genome-wide expression profiling of exogenous SPARC-processed HBVSMCs, which was then intersected with mitochondria-related genes in the GO database. We identified 40 mitochondria-related DEGs in SPARC-induced HBVSMCs, of which the down-regulated HK2 is particularly prominent. The results of PPI and GO analysis showed that HK2 was involved in multiple important signaling pathways. SPARC induced mitochondrial damage and down-regulated HK2 expression in HBVSMCs. Interference with HK2 expression induced apoptosis of HBVSMCs and decreased MMP, indicating the involvement of HK2 in SPARC-induced mitochondrial pathway apoptosis of HBVSMCs.

The biological mechanism of IA formation is highly complex. A recent meta-analysis (Jabbarli et al., 2020) identified important markers associated with functional pathways through analyzing more than 350 diagnostic markers of the IA wall. The results showed that cell adhesion, inflammation, apoptosis and oxidative stress are mainly concerned with the pathophysiological process of IA formation, and some other pathways such as calcium ion channel, lipid and glucose metabolism, cytoskeletal regulation, hormone pathway, especially estrogen metabolism pathway, etc., also exist. The apoptosis of smooth muscle cells is particularly prominent during the formation of IA, and the proportion of VSMC apoptosis is even higher in ruptured IA. A recent single-cell sequencing analysis of mouse IA models highlighted the fundamental link between the occurrence of IA and mitochondrial dysfunction, which can lead to chronic organelle damage and activation of apoptotic pathways (Martinez et al., 2022; Chen et al., 2023). This means that the functional state of smooth muscle is a major component in maintaining blood wall strength and homeostasis, and that an in-depth exploration of factors affecting mitochondrial function status of VSMC may be a potential target for intervention in IA.

Secreted protein acidic and rich in cysteine (SPARC) is a multifunctional glycoprotein (Yan and Sage, 1999; Pascual-Pasto et al., 2022) functionally characterized by anti-adhesion and anti-proliferation (Yan and Sage, 1999). It can regulate the extracellular matrix by binding to structural matrix proteins and eliminating adhesion (Casillas and Erickson, 1975; Wong and Sukkar, 2017). Currently, SPARC is widely studied in the field of oncology, and it has also attracted increasing attention in vascular diseases. Studies on a mouse model of pulmonary hypertension under hypoxia conditions showed that TGF-β1 (Bai et al., 2021) or hypoxia-inducing factor 2α (HIF2α) signaling pathway induces the expression of SPARC in human pulmonary artery smooth muscle cells (PASMCs). SPARC secreted by pulmonary artery endothelial cells triggers proliferation of PASMCs (Veith et al., 2022). In addition, single-cell sequencing of human abdominal aortic aneurysms suggested that SPARC was highly expressed in proliferation-associated smooth muscle cells and fibroblasts. Previous studies have confirmed that SPARC is also co-expressed in IA and is related to metalloproteinases. These studies provide a new perspective on the pathogenesis of IA. SPARC may regulate ECM and smooth muscle cell function, and participate in the occurrence of IA.

Our data showed that SPARC treatment increased apoptosis. The mitochondrial ultrastructure observed under the transmission electron microscopy showed that SPARC could impair the integrity of HBVSMC mitochondrial structure, which manifested as mitochondrial swelling, mitochondrial ridge breakage or even disappearance, and unclear mitochondrial membrane gap. Moreover, JC-1 staining indicated the decrease of mitochondrial membrane potential. Decreased MMP is one of the early manifestations of apoptosis (Zhang et al., 2017; Zorova et al., 2018), which suggested that SPARC may induce smooth muscle cell apoptosis through mitochondrial pathway.

In order to further clarify the downstream target of SPARC-induced apoptosis, we sequenced the whole genome of SPARC-treated HBVSMCs and applied bioinformatics methods to filter differential genes related to mitochondrial function. Our results indicated that TIMM9, STK24, and HK2 were significantly different compared to the normal group, and the results of functional enrichment analysis also supported that these gene played certain roles in mitochondrial membranes and stroma. The PPI network reflected the interactions between DEMRGs and we identified 10 hub genes, including TOMM40, GRPEL1, GRPEL2, TIMM8A, TIMM9, HSP90AA1, VDAC2, MCL1, HK2, and CPT2. Translocase of inner mitochondrial membrane (TIMM9) is a mitochondrial intermembrane chaperone involved in multi-channel transmembrane protein introduction into the inner mitochondrial membrane (Porcu et al., 2014). Translocase of outer mitochondrial membrane 40 homolog (TOMM40) is a subunit formed by translocation enzymes in the mitochondrial outer membrane and is critical for transporting proteins to the mitochondria and for maintaining normal mitochondrial function. This suggests that decreased TOMM40 may mediate hepatocyte apoptosis (Wang et al., 2018, 2019). GrpEL1 is a nucleotide exchange factor that assists mtHSP70 in non-naturally folded proteins in mitochondria. Increasing GrpEL1 can promote the binding of GrpEL1 and mtHSP70, thereby stimulating neuronal mitochondrial homeostasis (Ma et al., 2022). Both GrpEL1 and GrpEL2 bind to mtHsp70 as heterooligomeric subcomplexes and regulate the function of mtHsp70 (Srivastava et al., 2017). GrpE-like 2 (GrpEL2), a nucleotide exchange factor, has been shown to regulate the mitochondrial input process to maintain mitochondrial homeostasis, and overexpression of Grpel2 can alleviate apoptosis and mitochondrial dysfunction (Yang et al., 2023). In addition, inhibition of HSP90AA1 expression promotes cell apoptosis via modulating expression of genes related to cell cycle and apoptosis (Chu et al., 2013; Yang et al., 2022). Apart from TIMM9, these hub genes were down-regulated in the SPARC-treated group, which further confirmed that SPARC induced mitochondrial dysfunction of HBVMCs.

Hexokinase 2 (HK2) is the first rate-limiting enzyme in the glycolytic pathway and plays an important role in cell survival. Studies (Ahmad et al., 2002; Yao et al., 2014; Roberts and Miyamoto, 2015) demonstrated that HK2 can inhibit the apoptosis of mitochondrial pathway by maintaining the integrity of mitochondrial structure and function and inhibiting oxidative stress, thus playing a protective role in cells. Specifically, HK2 can bind to mitochondrial voltage-dependent anion channel (VDAC) in the outer membrane of mitochondria to form HK2-VDAC complex, which reduces the release of Cyt-C and protects mitochondrial function (Mathupala et al., 2006; Shoshan-Barmatz et al., 2015). Disassociation of HK2 from mitochondria elicits cell death (Chiara et al., 2008). A latest study found that mitochondrial HK2 prevents mitochondrial translocation of BAD, BAX proteins and activation of caspase-3, thereby alleviating drug-induced pyroptosis of cancer cells (Cai et al., 2021).

Secreted protein acidic and rich in cysteine (SPARC) can regulate glucose metabolism in tumor cells, and such a process is related to HK2. SPARC negatively regulates the expression of HK2 to affect the glycolysis process of cells. Studies (Hua et al., 2015) indicated that SPARC participates in the glycolysis process of liver cancer cells by down-regulating the expression of HK2. In addition, SPARC shows dose-dependent inhibition of HK2 activity and decreased mitochondrial activity on human and mouse ovarian cancer cell lines, which is related to the energy metabolism of tumor metastasis (Naczki et al., 2018). Metabolic regulation of vascular diseases has received extensive attention. Deficiency of prohibitin 2 enhances glycolysis in VSMC mediated by increased pyruvate kinase (PKM2) expression and inhibits the contractile phenotype (Jia et al., 2022). HnRNPA1 is a key regulator of PKM mRNA splicing, and platelet-released TGF promotes glucose metabolism of PASMCs and pulmonary vascular remodeling by up-regulating PKM2 expression through regulating hnRNPA1 (Zhu et al., 2022). Our study was the first time to reveal the key role of SPARC mediating mitochondrial HK2 in HBVSMC. SPARC treatment decreased mitochondrial membrane potential and increased smooth muscle apoptosis. After the knockdown and overexpression of HK2, it was further confirmed that SPARC acting on HK2 caused mitochondrial dysfunction and damage to HBVSMCs, and this process may be involved in the formation of IA. However, whether SPARC can affect the glucose metabolism of HBVSMC through HK2 should be further studied in the future.

Secreted protein acidic and rich in cysteine (SPARC) expression in VSMCs has received widespread attention as a possible target for regulating vascular homeostasis (Motamed et al., 2002; Veith et al., 2022). Previous studies have confirmed that both SPARC mRNA and protein expression are significantly upregulated in IA specimens (Li et al., 2013). Moreover, SPARC in IA has been correlated with expression of matrix metalloproteinases, suggesting that there may be a potential link between SPARC and IA (Ye et al., 2016). VSMCs constitute a major supporting part of the vascular wall, and their growth or programmed apoptosis plays an important role in the strength and morphology of blood vessels (Zhao et al., 2018; Frosen et al., 2019). Single-cell sequencing of mouse IA further revealed significantly higher levels of mitochondrial dysfunction and necrotic apoptosis in Mo/MΦ and VSMCs (Chen et al., 2023). This indicates that mitochondria-induced necrotic apoptosis is involved in IA formation. In this study, we confirm *in vitro* that SPARC induces apoptosis in the mitochondrial pathway of smooth muscle cells by down-regulating HK2, and hypothesize that SPARC may promote the progression of IA and be a novel target for IA intervention. However, further studies are needed to validate these results.

There are some limitations to our study. First, due to the small sample size and large tissue variability in the IA datasets, there may be a bias in the assessment of SPARC expression with respect to IA formation and rupture risk. Secondly, this study explored the downstream targets HK2 of mitochondrial dysfunction in VSMC after SPARC treatment. The specific regulatory mechanisms of HK2 and mitochondria still need to be explored more deeply. In addition, this study only includes *in vitro* cellular experiments, and it is necessary to construct IA animal models to improve *in vivo* experiments.

## Conclusion

Our study provides a new target for SPARC in the pathogenesis of IA. In addition, the study highlights a previously unrecognized function of mitochondrial HK2 in regulating smooth muscle cell death.

## Data availability statement

The datasets presented in this study can be found in online repositories. The names of the repository/repositories and accession number(s) can be found in this article/Supplementary material.

## Ethics statement

The studies involving humans were approved by the Ethics Committee on Scientific Research of Shandong University Qilu Hospital. The studies were conducted in accordance with the local legislation and institutional requirements. The participants provided their written informed consent to participate in this study.

## Authors contributions

DZ: Data curation, Formal analysis, Visualization, Writing – original draft, Writing – review and editing. TL: Data curation, Formal analysis, Funding acquisition, Methodology, Writing – original draft, Writing – review and editing. XT: Formal analysis, Methodology, Writing – review and editing. CY: Conceptualization, Data curation, Formal analysis, Methodology, Writing – original draft. PJ: Software, Supervision, Visualization, Writing – review and editing. TZ: Investigation, Supervision, Writing – review and editing. HK: Funding acquisition, Project administration, Writing – review and editing. YW: Funding acquisition, Project administration, Supervision, Writing – review and editing.

## Footnotes

1
www.gsea-msigdb.org

2
http://string-db.org/

3
https://www.sangerbox.com/
