# Supplementary material for: Secreted protein acidic and rich in cysteine (SPARC) induces apoptosis of human brain vascular smooth muscle cells through regulating HK2 in intracranial aneurysm
Source: Front Mol Neurosci. 2023 Nov 23;16:1290556. doi: 10.3389/fnmol.2023.1290556 (PMC10702226; doi:10.3389/fnmol.2023.1290556)
Supplement: Supplementary file 1 [file Table_1.docx]

Supplementary Table S1 siRNA used in this study

| siRNA | Sense(5'-3') |
| --- | --- |
| siRNA HK2-1# | Forward (5'-3'): GCAGAAGGUUGACCAGUAUTT  Reverse (5'-3'): AUACUGGUCAACCUUCUGCTT |
| siRNA HK2-2# | Forward (5'-3'): CCAGAAGACAUUAGAGCAUTT  Reverse (5'-3'): AUGCUCUAAUGUCUUCUGGTT |
| siRNA HK2-3# | Forward (5'-3'): CCUGCAACACUUAGGGCUUTT  Reverse (5'-3'): AAGCCCUAAGUGUUGCAGGTT |
| NC | Forward (5'-3'): UUCUCCGAACGUGUCACGUTT  Reverse (5'-3'): ACGUGACACGUUCGGAGAATT |
