# Supplementary material for: Secreted protein acidic and rich in cysteine (SPARC) induces apoptosis of human brain vascular smooth muscle cells through regulating HK2 in intracranial aneurysm
Source: Front Mol Neurosci. 2023 Nov 23;16:1290556. doi: 10.3389/fnmol.2023.1290556 (PMC10702226; doi:10.3389/fnmol.2023.1290556)
Supplement: Supplementary file 2 [file Table_2.docx]

| Supplementary Table S2. Antibodies information used in this study | | |
| --- | --- | --- |
| Antibodies | SOURCE | IDENTIFIER |
| HK2 | Proteintech | 22029-1-AP |
| SPARC | Proteintech | 15274-1-AP |
| Bax | Proteintech | 50599-2-IG |
| Bcl-2 | Proteintech | 26593-1-AP |
| Cleaved PARP | Cell Signaling Technology | 5625 |
| GADPH | Goodhere | AB-P-R001 |
