# Supplementary material for: Secreted protein acidic and rich in cysteine (SPARC) induces apoptosis of human brain vascular smooth muscle cells through regulating HK2 in intracranial aneurysm
Source: Front Mol Neurosci. 2023 Nov 23;16:1290556. doi: 10.3389/fnmol.2023.1290556 (PMC10702226; doi:10.3389/fnmol.2023.1290556)
Supplement: Supplementary file 3 [file Data_Sheet_1.docx]

**GSE15629 and GSE122897 analysis**

**#GEOquery**

library(GEOquery)

gse_number = "GSE15629/GSE122897"

eSet <- getGEO(gse_number,

destdir = '.',

getGPL = F)

eSet = eSet[[1]]

exp <- exprs(eSet)

boxplot(exp)

pd <- pData(eSet)

p = identical(rownames(pd),colnames(exp));p

if(!p) exp = exp[,match(rownames(pd),colnames(exp))]

gpl_number <- eSet@annotation

library(stringr)

k1=str_detect(pd$title,"temporal")

k2=str_detect(pd$title,"Unruptured")

Group=ifelse(k1,"Control",ifelse(k2,"UIA","RIA")

Group=ifelse(str_detect(pd$title,"temporal"),"Control","IA")

Group = factor(Group,

levels = c("Control","UIA","RIA"))

library(GEOquery)

a = getGEO(gpl_number,destdir = ".")

b = a@dataTable@table

colnames(b)

ids = b[,c("ID","Gene Symbol")]

colnames(ids) = c("probe_id","symbol")

ids = ids[ids$symbol!="" & !str_detect(ids$symbol,"///"),]

**#Boxplot**

library(ggplot2)

p = ggplot(SPARC_express, aes(x=Group, y=expression, color=Group)) +

geom_boxplot()

p + geom_jitter(shape=16, position = position_jitter(0.2))

p + scale_color_manual(values = c("#efc000", "#4dbbd5", "#e64b35"))

**#ROC curve**

library(tidyverse)

library(pROC)

library(ggplot2)

exp_SPARC <- read.table("~/file.txt", header = T)

exp_SPARC$outcome <- factor(exp_SPARC$outcome, levels = c("Control", "IA/UIA/RIA"))

head(data)

# outcome SPARC

# 1 Control 8259

# 2 Control 5511

# 3 Control 7038

# 4 Control 4184

# 5 Control 23384

# 6 Control 16513

SPARC_roc<- roc(data$outcome, data$SPARC)

plot(SPARC_roc, col="red/blue",#颜色

legacy.axes=T,

print.auc=TRUE,

print.thres=TRUE,

grid=c(0.2,0.2),grid.col="grey")

**RNA-seq analysis**

**#Differential analysis**

library(limma)

design=model.matrix(~Group)

fit=lmFit(exp_seq,design)

fit=eBayes(fit)

deg_seq=topTable(fit,coef=2,number = Inf)

deg_seq <- mutate(deg_seq,probe_id=rownames(deg_seq))

logFC_t=0.585

P.Value_t = 0.05

k1 = (deg$P.Value < P.Value_t)&(deg$logFC < -logFC_t)

k2 = (deg$P.Value < P.Value_t)&(deg$logFC > logFC_t)

deg_seq <- mutate(deg_seq,change = ifelse(k1,"down",ifelse(k2,"up","stable")))

**#Volcano map**

Volcano_plot <- ggplot (data = deg_seq,

aes(x = log(Fold Change),

y = -log10(P.Value))) +

geom_point(alpha=0.4, size=3.5,

aes(color=change)) +

ylab("-log10(Pvalue)")+

scale_color_manual(values=c("green", "black","red"))+

geom_vline(xintercept=c(-logFC_t,logFC_t),lty=4,col="black",lwd=0.8) +

geom_hline(yintercept = -log10(P.Value_t),lty=4,col="black",lwd=0.8) +

theme_bw()

Volcano_plot

**#Heatmap**

cg = deg_seq$symbol[deg_seq$change !="stable"]

n=exp_seq[cg,]

library(pheatmap)

annotation_col=data.frame(group=Group)

rownames(annotation_col)=colnames(n)

heatmap_plot <- pheatmap(n,show_rownames = T,

scale = "row",

shown_colnames= F,

cluster_cols = F,

annotation_col=annotation_col,

breaks = seq(-2,2,length.out = 100)

)

heatmap_plot
